# Revisiting the link between cognitive decline and masticatory dysfunction

**DOI:** 10.1186/s12877-017-0693-z

**Published:** 2018-01-05

**Authors:** Chia-shu Lin

**Affiliations:** Department of Dentistry, School of Dentistry, National Yang-Ming University, No. 155, Sec. 2, Linong Street, Taipei, 11221 Taiwan

## Abstract

Age-related decline in cognitive functions and dementia are major challenges in geriatric healthcare. Accumulating evidence from clinical, epidemiological and animal research suggests that tooth loss may be a risk factor for the decline of cognitive functions. This issue highlights the role of the brain-stomatognathic axis in geriatric medicine. Whether input from the stomatognathic apparatus can affect the brain remains an open debate. By revisiting the evidence published in the past five years, we argue that the hypothesis regarding the association between cognitive decline and masticatory dysfunction should be carefully interpreted. Most of the available clinical and epidemiological studies present only cross-sectional data. With respect to the prospective studies, important confounding factors, such as nutritional and physical conditions, were not fully controlled for. Animal research has revealed that hippocampal deficits may play key roles in the observed cognitive decline. However, experimental intervention and outcome assessments may not capture the condition of human subjects. Brain neuroimaging research may be suitable for bridging the gap between clinical and animal research, potentially contributing to (a) the clarification of the brain network associated with mastication, (b) the identification of brain imaging markers for exploring the mechanisms underlying long-term changes in masticatory functions, and (c) the elucidation of interactions between mastication and other cognitive-affective processing systems. Three potential models of the brain-stomatognathic axis and relevant hypotheses are summarized, focusing on the sensory feedback mechanisms, the compensation of motor control, and cerebellar deficits. Finally, we highlight four critical aspects of study and experimental design that should be considered in future research: (a) the refinement of the considered behavioral assessments, (b) the inclusion of baseline changes in mental and physical conditions, (c) a prospective experimental design with longitudinal observations, and (d) a precise determination of the effect size of the association between cognitive decline and masticatory dysfunction.

**Electronic supplementary material:**

The online version of this article (10.1186/s12877-017-0693-z) contains supplementary material, which is available to authorized users.

## Background

Does tooth loss increase the risk of dementia? Can improvements in chewing ability prevent cognitive impairment or ameliorate cognitive decline? The association between the brain and the stomatognathic system, which plays a key role in chewing and swallowing [1], has recently been hotly debated in the media [2–5]. Behind these arguments is the emerging concept of the ‘brain-stomatognathic axis’, generally defined as a complex communication network between the brain, including both cortical and subcortical structures, and the stomatognathic system. The top-down control from the brain to the stomatognathic system, such as the coordination between jaw motion and tongue movement, has been established [1, 6]. However, what has remained unclear is whether input from peripheral structures, such as the sensory signals from the jaw and teeth, can likewise affect the brain. Aging is associated with a decline in both stomatognathic (e.g., tooth loss) [7] and brain functions (e.g., cognitive impairment or dementia) [8]. Therefore, the mechanisms underlying the brain-stomatognathic axis have emerged as critical issues in neuroscience as well as in orofacial and geriatric medicine.

Accumulating evidence suggests that cognitive decline may be associated with masticatory dysfunction [9–15]. The term ‘cognitive decline’ generally refers to the decreased cognitive abilities, including short-term and long-term memory, reasoning, and language abilities, which can be associated with normal aging or dementia [16]. ‘Masticatory dysfunction’, as an umbrella term, refers to a debilitating condition in which normal masticatory function is compromised due to structural factors (e.g., tooth loss) or functional factors (e.g., weaker biting force or poorer masticatory performance) [17]. The association between cognitive decline and masticatory dysfunction was highlighted in the famous Nun study, which revealed that the number of missing teeth was associated with an increased risk of dementia [18]. The conclusion was supported by recent meta-analytical findings [9] and evidence from clinical [19] and animal research [10]. These data all suggested a close association between cognitive decline and masticatory dysfunction. For the general public, it is tempting to think that improving masticatory function may be a new path for preventing or ameliorating cognitive decline in elderly individuals [2–5].

In this review, we argue that the current evidence has not provided adequate information regarding the mechanisms underlying the relationship between cognitive decline and masticatory dysfunction. These findings may suggest a stomatognathic-to-brain effect, i.e., poorer masticatory conditions predispose individuals to cognitive decline. However, the cause-and-effect relationship is unclear, and a gap remains between theoretical and clinical investigations. To assess this issue, we first systematically reviewed the recent evidence in the past five years (2012.10.15 ~ 2017.10.15) regarding the association between cognitive decline and masticatory dysfunction, focusing on the findings from clinical/epidemiological and animal research (for a comprehensive review of this topic based on earlier publications, please see [15, 20]). The limitations of these research models will be discussed. Second, we reviewed the evidence from brain neuroimaging studies. We propose that neuroimaging would be an ideal tool for bridging the gap between clinical/epidemiological evidence and animal research. Third, we summarized and put forward three hypotheses regarding the mechanisms underlying the brain-stomatognathic axis. These hypotheses can be tested using neuroimaging methods. Finally, we summarized the future directions for research into the brain-stomatognathic axis.

### Evidence from clinical and epidemiological research

First, we reviewed the findings from systematic reviews or meta-analyses published in the past five years regarding the association between cognitive decline and masticatory dysfunction (for detailed procedures of the literature search and screening, please see Additional file 1). As shown in Table 1, two studies supported for the notion that decreased masticatory function is associated with diminished cognitive functions [9, 11], whereas two studies concluded that the association remained ‘inconclusive’ or ‘unclear’ [12, 13]. Another study showed no statistically significant difference in the number of teeth between elderly individuals with and without dementia [14]. It should be noted that all five of the reviews stated some potential confounding effects or between-study heterogeneity in study design [9, 11–14] As a cause-and-effect relationship cannot be inferred from the findings of the cross-sectional studies [11, 13], we restrict our discussion to studies with a longitudinal, prospective design. In total, 13 prospective studies ([21–33]) were reported on by the five reviews [9, 11–14]. Nine studies reported that a worse dental status was associated with cognitive decline ([22, 25, 27–29, 32, 33], one study showed a borderline significance of the association [31]), three studies reported no significant association [24, 26, 30], and one reported a negative association, i.e., more missing teeth was associated with a lower risk of dementia [21]. Though the majority of the studies revealed an association between cognitive decline and masticatory dysfunction, a great heterogeneity existed in the outcome assessment regarding masticatory function. For example, the number of missing teeth was adopted as a primary index related to masticatory function [17]. In one study, the condition of tooth loss was categorized based on the number of teeth (fewer than 20 or not) [30], while another study assessed the number of missing teeth per decade of follow up [25].

**Table 1:** Conclusion from the systematic review and meta-analysis on clinical and epidemiological research published in the past five years

Search criteria: 1. Key word combination: (chewing OR masticat* OR “tooth loss” OR “teeth loss” OR “number of teeth”) AND (cognit* OR memory OR dement*) AND (“systematic review” OR meta-analysis) 2. Language: English 3. Publication date: 2012.10.15–2017.10.15			
Reference	Criteria of study selection	Number of studies included	Major findings or conclusion (direct quotation)
Tonsekar et al. 2017 [13]	Publications on the relation between periodontitis, tooth loss and dementia	Total: 8^a^ PT:4 RT:3	‘The literature on chronic periodontitis and multiple tooth loss as risk factors to dementia remains inconclusive.’
Tada and Miura 2017 [11]	Publications that assessed associations between mastication and cognitive function, cognitive decline and dementia among population over 40 years old	Total: 33 CS:22 PT:11	‘Most studies point to a positive association between mastication and cognitive function, including dementia among elderly people.’
Wu et al. 2016 [12]	Publications that examined the effect of oral health on change in cognitive health or dementia incidence, or the publications that examined the reverse effect.	Total: 11^b^ (all longitudinal studies)	‘Similarly, cognitive decline was not consistently associated with greater loss of teeth or number of decayed teeth.’
Delwel et al. 2016 [14]	Publications about oral health and orofacial pain Comparison was made between the older people with and without dementia.	Total: 19^c^ CS: 9 CC: 3 RCT: 1 Longitudinal: 6	‘……they had an equivalent number of teeth present, similar rate of edentulousness, and equivalent decayed missing filled teeth index.’
Cerutti- Kopplin et al. 2016 [9]	Publications on the association between oral health and cognitive function, via prospective cohort study designs	Total: 10 PT: 10	‘Within the limits of the quality of published evidence, this meta-analysis lends further support to the hypothesis that tooth loss is associated with an increased risk of cognitive impairment and dementia.’

*CC* Case-control studies, *CS* cross-sectional studies, *PT* prospective studies, *RCT* randomized controlled trials, *RT* retrospective studies

^a^Only the studies of human subjects

^b^Only the studies using the data from oral health status to predict cognitive status

^c^Only the studies that reported the number of present teeth

Second, it is noteworthy that while most of the studies showed an association between tooth loss and cognitive decline, the elderly participants may have undergone long-term adaptation to their edentulousness/tooth-loss condition during chewing. Therefore, though being a feasible and reliable index, the number of teeth lost may not fully capture the change in masticatory function, and functional assessments (e.g., masticatory performance) may provide a better index for masticatory dysfunction [11]. To understand the association between masticatory performance and cognitive conditions, we performed a review by systematically searching for and screening the original studies that directly investigated the association between cognitive decline and masticatory ability using a masticatory performance test (Table 2). We found five studies published in the past five years that objectively quantified masticatory performance using functional assessments, including the color-changeable chewing gum test [34, 35], the two-color chewing gum test [36, 37], and the Optocal chewing test and the sieve fractionation test [38]. Masticatory performance decreased in patients with dementia compared to the controls [36, 38] and was associated with performance on cognitive tests [34, 35, 37] (Table 2). These findings suggested that functional assessments may be useful for assessing practical chewing performance.

**Table 2:** Findings from the clinical/epidemiological research that objectively quantified masticatory performance using functional assessments published in the past five years

Search criteria: 1. Key word combination: (“mixing ability” OR “cutting ability” OR “crushing ability” OR “masticatory ability” OR “chewing ability” OR “masticatory performance” OR “masticatory efficiency” OR “chewing efficiency” OR “chewing performance”) AND (cognit* OR memory OR dement*) AND (cognit* OR memory OR dement*) 2. Language: English 3. Publication date: 2012.10.15–2017.10.15			
Reference	Study group	Outcome assessment	Major findings (direct quotation)
Kim et al. 2017 [34]	295 participants (age ≥ 70 years), a rural city of Korea	Color- changeable chewing gum, MMSE-DS, ADL, MNA	‘Our findings suggest that poor chewing ability is associated with cognitive impairment or dementia in the elderly living in rural area.’
Campos et al. 2017 [38]	16 AD patients (mean age = 76.7yers) and 16 controls (mean age = 75.2 years)	Optocal chewable test, sieve fractionation test, MMSE	‘Compared to controls, mild AD patients had decreased MP (*P* < 0.01) and MMSE (*P* = 0.01). MP showed a moderate negative correlation with MMSE (*r* = −0.69).’
Weijenberg et al. 2015 [37]	114 patients with dementia (age 66–97 years)	Two-color gum mixing ability test, a multi-domain neuropsychological test battery	‘Significant relationships were observed between masticatory performance and general cognition and between masticatory performance and verbal fluency.’
Elsig et al. 2015 [36]	29 patients with dementia (age ≥ 75 years), 22 controls^a^	Two-color mixing test, dental and nutritional assessment	‘The chewing efficiency by visual inspection proved worse in participants with dementia than in the controls (*p* < 0.011) and explained 9.3% of the variance in the diagnosis of dementia.’
Kimura et al. 2013 [35]	269 community-dwelling elderly aged ≥75 living in Tosa, Japan	Color-changeable chewing gum, MMSE, HDSR and FAB, ADL, QOL, FDSK-11	‘Lower cognitive functions were significantly related to low chewing ability; MMSE (*P* = 0.022), HDSR (*P* = 0.017) and FAB (*P* = 0.002).’

*AD* Alzheimer’s disease, *ADL* activities of daily living, *DS* Dementia Screening, *FAB* Frontal Assessment Battery, *FDSK-11* Food Diversity Score Kyoto, *HDS-R* Hasegawa Dementia Scale-Revised, *MMSE* Mini-mental state examination, *MNA* Mini Nutritional Assessment, *MP* masticatory performance, *QOL* quality of life

^a^Including 19 cognitively normal participants and 3 patients with mild cognitive impairment

In general, the findings from recent reviews (Table 1) and studies of masticatory performance (Table 2) suggested that masticatory dysfunction was a potential risk factor for cognitive impairment in elderly individuals. However, these conclusions must be carefully interpreted due to the following limitations:

(A) As previously stated, there is a great heterogeneity among the methods for assessing masticatory function. In fact, only a small number of epidemiological surveys have directly assessed masticatory function, such as masticatory cutting ability, mixing ability or bite force, all of which represent different aspects of masticatory performance [17, 39]. Moreover, the self-reported chewing experience was not consistent with the results of masticatory performance assessments [40]. Therefore, it is questionable whether the number of teeth lost, a simplified epidemiological index of the dental condition, can fully capture the difficulty experienced during eating.

(B) Elderly individuals may develop some adaptive strategies to cope with tooth loss during eating. For example, they may carefully choose the type of food they eat and a method for processing it in order to more easily chew and swallow. Therefore, in regard to the association between cognitive decline and masticatory dysfunction, an individual’s nutritional status, eating habits and general physical condition are critical factors to be adjusted for, especially in a longitudinal study [9].

(C) It is noteworthy that not all of the clinical/epidemiological studies included baseline or follow-up assessments of cognitive functions. Therefore, long-term changes in cognitive functions, which can be partly explained by normal aging, may not be adequately evaluated [16]. In addition, exercise is regarded as a critical factor to both physical fitness and cognitive ability in elderly individuals [41]. Long-term follow up on these factors would help to control for the effects of the baseline changes in general physical conditions.

### Evidence from animal research

Compared to clinical and epidemiological studies, animal research provides benefits in examining the relevant mechanistic cause-effect relationships, e.g., the cellular or neurochemical changes associated with the studied behavioral deficits [42–60]. These findings from animal research published in the past five years are summarized in Table 3. In general, these studies have supported the hypothesis that cognitive decline is associated with masticatory dysfunction. These studies have demonstrated that cognitive decline is related to cellular and neurochemical change in the hippocampus, including decreased cellular proliferation [47, 48, 50], decreased levels of brain-derived neurotrophic factor [44, 47, 59], as well as increased nitrous oxide levels [55] and extracellular dopamine levels [52]. These findings suggested that the hippocampus-dependent deficits in learning and memory may contribute to the association between cognitive decline and masticatory dysfunction [10]. Importantly, using animal models, researchers were able to investigate the interactional effect between masticatory dysfunction and other factors, such as the type of diet [46, 59], environmental stimuli [47], and stress [52]. These factors can partially ameliorate the cognitive deficits induced by masticatory dysfunction [46, 47]. These findings imply that masticatory dysfunction per se may not be the only determinant to cognitive decline. Rather, the interaction of this dysfunction with other factors can partially account for the observed cognitive deficits.

**Table 3:** Findings from the animal research published in the past five years

Search criteria: 1. Key word combination: (chewing OR masticat* OR “tooth loss” OR “teeth loss”) AND (hippocamp* OR parahippcamp* OR limbic) 2. Language: English 3. Publication date: 2012.10.15–2017.10.15			
Reference	Strain/Experimental manipulation	Behavioral findings	Cellular /neurochemical findings
Fukushima-Nakayama et al. 2017 [44]	C57BL/6 J mice/Normal (N) or solid (S) diet	Passive avoidance test (−) and Object location test (−) in S group	Hippocampal neurons, neurogenesis, neuronal activity (−), BDNF expression (−) in S group
Kubo et al. 2017 [48]	SAMP8 mice/Tooth loss soon after tooth eruption	Morris water maze (−)	Cell proliferation/cell survival in DG (−) Synaptophysin expression in HIP (−) Newborn cell differentiation in DG (X)
Avivi-Arber et al. 2016 [43]	7 strains of female mice/Molar extraction		Regional and voxel-wise volumes of cortical brain regions (−) and subcortical, sensorimotor, temporal limbic regions (+)
Takeda et al. 2016 [59]	C57BL/6 J mice/Molar extraction (E) and powder (P) or solid (S) diet	Step-through passive avoidance test (−) for E/S and E/P groups 16 weeks later	BDNF-related mRNA in HIP (−) for E/S and E/P groups 16 weeks later
Oue et al. 2016 [53]	APP transgenic mice/Molar extraction	Step-through passive avoidance test (X)	Amount of Aβ and number of pyramidal cells in HIP (X)
Kondo et al. 2016 [47]	SAMP8 mice/Molar extraction (E) or intact (I) and standard (S) or enriched (R) environment	Morris water maze (−) in E group	Proliferation and survival of newborn cells in DG (−) and BDNF levels in HIP (−) in E group
		The effect was attenuated in E/R group	
Kida et al. 2015 [46]	Tooth extraction (E) and zinc-deficient (ZD) or zinc-sufficient (ZS)	Spatial memory (−) in E/ZD group; recovered in E/ZS group	Astrocytic density in CA1 (+) in ZD group
Pang et al. 2015 [55]	KM mice/Molar extraction	Morris water maze (−)	Levels of NO and inducible nitric oxide synthase in HIP (+)
Ono et al. 2015 [52]	Stress condition (S), stress with voluntary chewing condition (SC), and control condition (C)	Open-Field Test and Elevated Plus Maze Test (+) in SC vs. S	Extracellular dopamine concentration in HIP (+) in SC vs. S
Su et al. 2014 [57]	CD1 mice/Molar extraction		Density and absorbance of doublecortin- and neuronal nuclear antigen-positive cells (−)
Okihara et al. 2014 [51]	C57BL/6 J mice/Chow diet (C) or liquid diet (L)	Passive avoidance test (−) in L vs. C	BDNF level (+), TrkB (−), and number of pyramidal neurons (−) in HIP in L vs. C
Utsugi et al. 2014 [60]	Hard (H) or soft (S) diet	Avoidance of butyric acid (−) in S vs. H group	Expression of Fos-ir cells at the Pr5 (+) and the density of BrdU-ir cells in SVZ and OB (+) in H vs. S group
		In S group, avoidance of butyric acid and responses to odors and neurogenesis in SVZ were reversed after hard-dieting for 3 months	
Nose-Ishibashi et al. 2014 [50]	C57BL6/J mice/Post-weaning and Hard (H) or soft (S) diet	Home cage activity (−), open fielf test (+), prepulse inhibition (−), learning and memory tests (X) in S vs. H group	Cell proliferation, BDNF and Akt1 gene expression (−) in HIP in S vs. H group
Kawahata et al. 2014 [45]	SAMP8 mice/molar extraction (E) or intact (I)	open-field test (+), object-recognition test (−) and weight (−) in E vs. I group	
Patten et al. 2013 [56]	Sprague-Dawley rats/Solid diet (S) or liquid diet (L)		Neuronal differentiation and survival (X), HPA-axis function (X), cell proliferation in HIP and hypothalamus (−) in L vs. S group
Niijima-Yaoita et al. 2013 [49]	Powdered (P) or standard (S) diet	Social interaction time (+)	Dopamine turnover (+) and D4 receptor expression (−) in frontal cortex in P vs. S group
Oue et al. 2013 [54]	Transgenic mice/molar extraction (E) or intact (I)	Passive avoidance test (−) in E vs. I group	Neuronal cell number in CA1/CA3 (−) and Aβ, Aβ40, and Aβ4 level (X) in E vs. I group
Akazawa et al. 2013 [42]	Hard (H) or normal (N) diet	Morris water maze (+) in H vs. N group	Expression of glutamate receptor 1 mRNA (+) in DG in H vs. N group

*APP* amyloid precursor protein, *BDNF* Brain-derived neurotrophic factor, *BrdU-ir* Bromodeoxyuridine-immunoreactive, *CA* cornua mmonis, *DG* dentate gyrus, *Fos-ir* Fos-immunoreacivity, *HIP* hippocampus, *HPA* hypothalamic-pituitary-adrenal, *NO* nitric oxide*, OB* olfactory bulb, *Pr5* the principal sensory trigeminal nucleus, *SAMP8* senescence-accelerated mouse prone 9, *SVZ* subventricular zone

Although animal research has provided a great deal of evidence regarding the mechanisms underlying the association between cognitive decline and masticatory dysfunction, several aspects need further clarification:

(A) One of the major challenges faced in interpreting these results is external validity, i.e., the extent to which we can generalize the findings from animal models to human subjects. Most of the animal studies adopted behavioral tasks, such as the Morris water maze and the passive avoidance task, which evaluated spatial and associative learning. One should bear in mind that a poor performance in these tasks does not necessarily reflect cognitive decline in elderly human subjects. The latter is a more complex condition, consisting of changes in short- and long-term memory, language, and reasoning.

(B) Most animal studies adopted tooth extraction as the experimental model to induce masticatory dysfunction, assuming that fewer molars leads to poorer chewing ability. In contrast, human subjects may develop adaptive strategies to cope with tooth loss. For instance, an earlier investigation on 315 edentulous elderly individuals showed that approximately 40% of them did not report chewing difficulty [61]. A recent investigation of elderly individuals revealed that chewing difficulty associated with a decreased number of functional units depended on the choice of food [62]. Notably, because experimental animals have a shorter lifespan, it may be difficult to evaluate the long-term adaptive effect of masticatory function using animal models.

### Bridging the gap: The role of brain neuroimaging in the investigation of the brain-stomatognathic axis

Neuroimaging methods, including structural and functional magnetic resonance imaging (MRI), are popular approaches for investigating oral sensory and motor functions. The structural and functional brain signatures associated with cognitive decline, either due to normal aging or dementia, have been widely studied [16]. Brain neuroimaging provides the advantage of directly assessing the brain-stomatognathic axis by recording changes in functional activation and structural signatures. Therefore, these tools are suitable for testing hypotheses based on previous neuroimaging and animal research findings. Here, we propose three ways in which neuroimaging research may significantly contribute our knowledge of the brain-stomatognathic axis.

#### Identifying the brain network associated with mastication

In an earlier study, Onozuka and colleagues reported an age-related difference in chewing-related brain activation. These authors found a consistent activation pattern between older and younger groups [63]. Critically, the findings revealed a core network of mastication, including the sensorimotor cortex, the thalamus, the cerebellum, and the frontal cortex, including the supplementary motor area and the premotor cortex [63, 64]. In general, such a network was consistently reported by other studies that adopted similar gum-chewing tasks [65–70] (Table 4). Neuroimaging studies further elucidated that both blood perfusion of the trigeminal nucleus and brain activation in somatosensory areas were associated with chewing-side preference [66, 71]. Higher masticatory performance was also associated with the individual intrinsic brain signatures, including the greater gray matter volume in the motor area and higher functional connectivity between the cortex and the cerebellum [72]. Critically, neuroimaging research revealed that not only regional brain activation but also functional connectivity in mastication-related networks were associated with chewing [73]. Moreover, the difference in the connectional pattern was related to age [74]. In general, these findings implied that the functional and structural differences in mastication-related networks may play a key role in the individual variation in masticatory functions.

**Table 4:** Findings from brain neuroimaging studies related to chewing published in the past five years

Search criteria: 1. Key word combination: (chewing OR masticat* OR “tooth loss” OR “teeth loss”) AND MRI AND brain 2. Language: English 3. Publication date: 2012.10.15–2017.10.15		
Reference	Task	MRI findings
Inamochi et al. 2017 [78]	Chewing, before (Day 0) and after (Day 1/Day 7) inserting a palatal plate	Decreased activation in the bilateral face S1/M1, putamen, left ACC, and right medial posterior frontal cortex on Day 1 vs. Day 0. Activation in the right S1/M1 and putamen recovered to Day 0 level by Day 7.
Choi et al. 2017 [65]	Gum chewing	Brain activations at the entorhinal cortex and the parahippocampal cortex, based on an region-of-interest analysis
Lotze et al. 2017 [67]	Rubberdam chewing	Increased activation at bilateral S1, S2, M1, PMC, SMA and CG, anterior CB, INS, OFC, THA and left pallidum
Lin et al. 2017 [74]	Resting (task-free) condition	The older subjects presented a different functional network associated with masticatory performance, compared to the younger subjects
Lin et al. 2015 [72]	Resting (task-free) condition	Grey matter volume in the motor and frontal regions, and the functional connectivity of the cerebellum, was associated with masticatory performance
Viggiano et al. 2015 [71]	After vs. before a mastication exercise (gum chewing)	Increased perfusion at the principal trigeminal nucleus but not in the dorsolateral-midbrain
Jiang et al. 2015 [66]	Rhythmic chewing	Increased activation at sensorimotor cortex contralateral to the chewing side of preference (CSP), midbrain and brainstem for left CSP, and CB for right CSP
Shoi et al. 2014 [70]	Gum chewing; full arch (with a removable partial denture, RPD) vs. short-dental arch (SDR)	Increased activation at middle FG, S1/M1, SMA, putamen, INS and CB for RPD group; no activation at middle FG in SDR group
Luraschi et al. 2013 [68]	Three task^a^, patients with a complete denture	(Across all three functional tasks) increased activation at bilateral pre (M1) and post (S1) central gyrus
Hirano et al. 2013 [81]	Two back-to-back ANT sessions with or without gum chewing	(During chewing) increased activation at anterior CG and left FG for the executive network; motor-related regions for attentional networks
Quintero et al. 2013a [69]	Gum chewing	Increased activation at CB, motor cortex, caudate nucleus, CG, and brainstem
Quintero et al. 2013b [73]	Gum chewing	Increased FC between bilateral M1 and S1, CB, CG and precuneus; increased FC between CB and contralateral CB, bilateral sensorimotor cortex, left superior temporal gyrus, and left CG
Yu et al. 2013 [82]	Stress induced by loud noise; gum-chewing	Anterior INS – dACC FC was increased by noise to a lesser extent during gum-chewing (vs. no gum-chewing)

*CB* the cerebellum, *CG* the cingulate gyrus, *dACC* the dorsal anterior cingulate cortex, *FC* functional connectivity, *FG* the frontal gyrus, *INS* the insula, *M1* the primary motor cortex, *OFC* orbitofrontal cortex, *PMC* the premotor cortex, *S1/S2* primary/secondary somatosensory cortex, *SMA* supplementary motor area, *THA* thalamus

^a^The tasks included lip pursing, jaw tapping and jaw clenching

#### Using brain imaging markers to explore the mechanisms underlying long-term changes in masticatory functions

The mastication-related brain regions identified in the studies discussed above can be adopted as imaging markers for better elucidating the mechanisms underlying long-term stomatognathic behavior. First, neuroimaging research helps us to understand the individual responses to prosthodontic treatment [75]. Earlier neuroimaging studies showed that during a clenching task, brain activation in the somatosensory area was associated with the wearing of an implant-supported overdenture (IOD) but not with the wearing of a complete denture (CD) [76]. Moreover, during a gum-chewing task, brain activation in the prefrontal cortex (PFC) was reduced in the IOD group compared to the CD group [77]. Furthermore, compared to the conventional removable partial denture, the patients wearing a shorten-dental-arch denture showed no activation at the middle frontal gyrus, corresponding to lower masticatory performance [70]. Second, neuroimaging research helps to explore the mechanisms underlying long-term adaptation of treatment. For example, after denture insertion, brain activation in the somatosensory cortex was significantly increased during a jaw-clenching task, corresponding to an increase in chewing efficiency [68]. When the participants adapted to the insertion of an experimental palatal plate, there was a consistent increase in the activation of sensorimotor regions [78]. These findings showed that differences in brain mechanisms may play critical roles in the individual variation in long-term adaptation to treatment, particularly for psychological adaptation (i.e., incorporating the denture as ‘part of the body’ [68].

#### Exploring the interaction between mastication and other cognitive-affective processing

Evidence from behavioral research has revealed that chewing is associated with changes in sustained attention and helps relieve stress [79]. Evidence from animal research has consistently revealed that mastication attenuates activity in the hypothalamic-pituitary-adrenal axis and reduces the effect of chronic stress [80]. Here, neuroimaging may help to bridge the gap between human behavioral and animal research. For example, chewing has been associated with an increased activation in the attentional network when the participants performed an attention-demanding cognitive task [81]. In contrast, chewing may attenuate stress-induced activation of the salience network [82]. In contrast to animal research, a great number of cognitive-affective tasks can be implemented during MRI scans, providing a direct and in situ assessment of the association between mastication and cognition.

In the following section, several potential mechanisms and relevant hypotheses are summarized and discussed.

### Hypotheses regarding the neural mechanisms underlying the brain-stomatognathic axis and recent supporting evidence

**Fig. 1:**
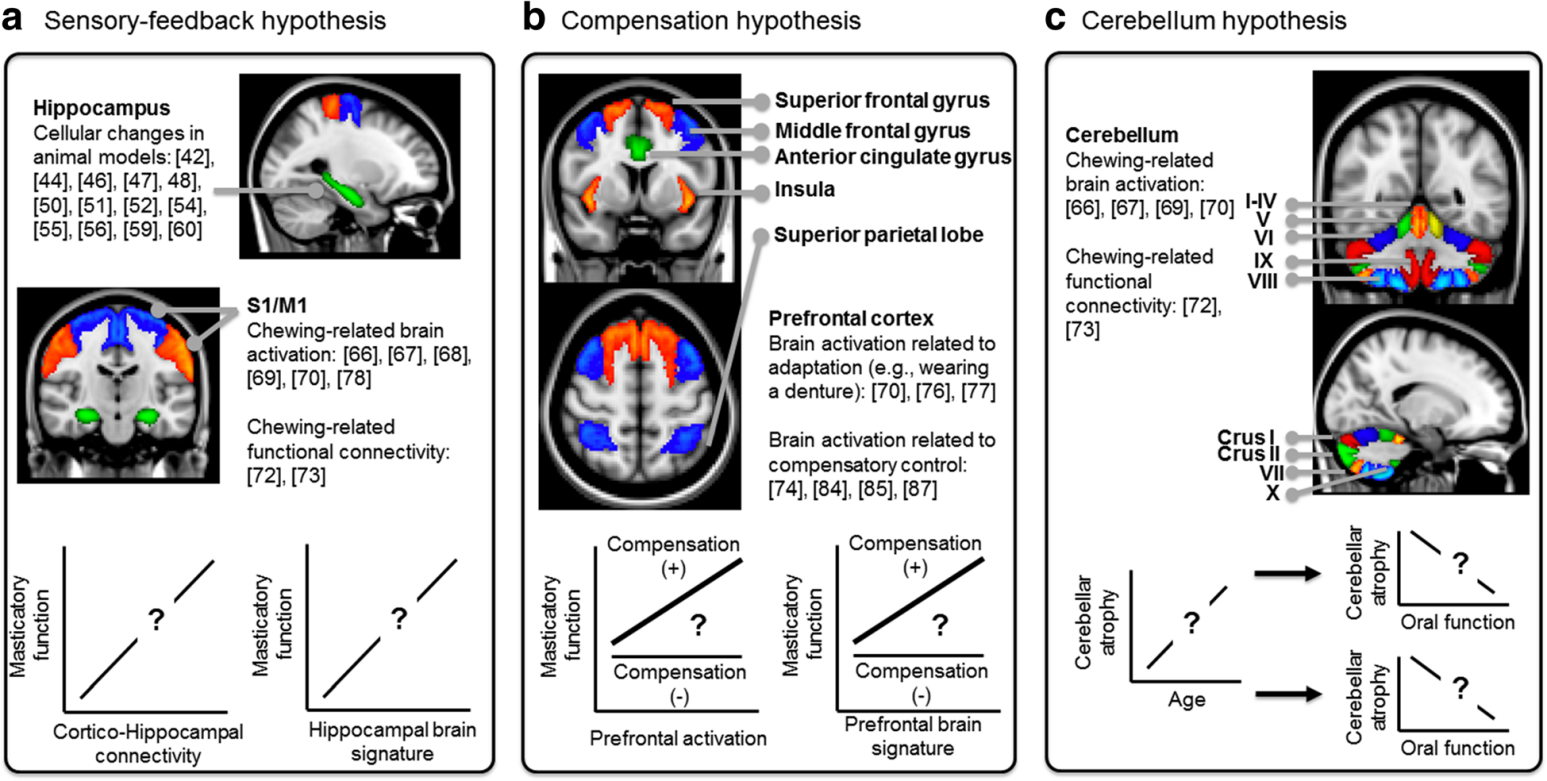
The potential mechanisms underlying the brain-stomatognathic axis and the relevant hypotheses. **a** According to the sensory-feedback hypothesis, the cortico-hippocampal connectivity between the sensorimotor cortex (S1/M1) and the hippocampus, which deficits were observed in animal research, would be associated with the masticatory functions. **b** According to the compensation hypothesis, the age-related compensatory motor control, primarily mandated by the PFC, may be compromised in some elderly people. The deficits in the PFC and the network of cognitive control would be associated with both cognitive decline and masticatory dysfunction. **c** According to the cerebellum hypothesis, the cerebellar deficits would separately influence both motor and cognitive abilities. Therefore, cerebellar atrophy or decreased cerebellar function would be associated with both cognitive decline and masticatory dysfunction. The number in the bracket refers to the cited research evidence related to the hypotheses

Current clinical / epidemiological findings have generally revealed a statistical correlation between cognitive decline and masticatory dysfunction. However, the neural mechanisms underlying this effect are still vague. Animal research has revealed that hippocampal deficits play a key role in this association. Based on the current evidence, we summarized and put forward several hypotheses for future neuroimaging studies (Fig. 1). These hypotheses are respectively focused on (a) the sensory-feedback mechanisms of the stomatognathic system; (b) the compensatory control of movement from the brain; and (c) the role of the cerebellum, a brain region closely associated with sensorimotor and cognitive decline.

#### The sensory-feedback hypothesis

Mastication may directly affect the brain via the sensorimotor circuitry (Fig. 1a). A potential mechanism is that the sensory feedback during mastication – primarily from the peripheral sensory apparatus – may stimulate the brain, with the hippocampus being a key target of this effect [9, 15]. Supporting the hypothesis, neuroimaging evidence has consistently shown an increased activation in the sensorimotor area during a chewing task [66–70, 78] (Table 4). Increased brain activation in these regions is associated with the use of dentures, as shown by cross-sectional [70] and longitudinal research [68]. Furthermore, one recent study with a small sample size revealed that gum-chewing was associated with increased brain activation in the hippocampal / parahippocampal area [65], consistent with the findings from animal research [10]. Recent evidence has revealed a preferential connectivity between the hippocampus and the sensory cortex in mice. In contrast, a preferential connectivity between the hippocampus and the association cortex was found in humans subject [83]. These findings suggest that, sensory information can be rerouted to the association cortex before reaching the hippocampus in humans [83]. Critically, compared to younger participants, the older participants showed a stronger association between masticatory performance and intra-cortical connectivity [74]. These findings would suggest that intra-cortical and cortico-hippocampal connectivity, which signifies sensory-feedback processing, is correlated with clinical metrics of masticatory functions – a hypothesis that remains untested with neuroimaging methods.

#### The compensation hypothesis

Accumulating evidence suggests that aging is associated with compensatory motor control processing, including increased monitoring as well as heightened attention and cognitive modulation [84, 85]. Such compensatory mechanisms are closely related to the brain regions that are associated with cognitive control and sensory integration, including the PFC, the parietal lobe, the insula, and the cingulate cortex [84, 85]. The PFC showed a significant age-related atrophy [86], which plays a key role in cognitive decline [16]. Therefore, masticatory dysfunction may reflect a decreased capacity of compensation in motor control (Fig. 1b). The hypothesis was supported by neuroimaging findings from participants performed simple motor tasks. For example, younger individuals were able to maintain automatic and highly coordinated movement. In contrast, as age increased, the participants needed to pay more attention to monitoring and controlling their movement [85, 87]. In terms of rhythmic movement, cortical connections are associated with a stronger effort in sensory integration and monitoring of motor control, while cerebellar connections are associated with an automatic modulation of movement [87]. A recent neuroimaging study revealed an age-related difference in the functional connectome of the mastication-related brain network. In the older participants, higher masticatory performance was positively correlated with the strength of intra-cortical and cortical-subcortical connectivity. In contrast, in the younger participants, high masticatory performance was positively correlated with connectivity between the somatosensory cortices and the cerebellum [74]. Neuroimaging evidence has consistently shown that wearing dentures is associated with increased brain activation in the PFC [70, 76]. Wearing implant-supported dentures, which provide a better subjective adaptation than conventional dentures, was found to be associated with reduced activation in the PFC [77]. Together, these findings imply that when the PFC function is compromised, such as in the context of dementia, older individuals would exhibit poorer compensatory control over chewing movement, dampening their masticatory function.

#### The cerebellum hypothesis

The previous hypotheses presume that a direct association exists between cognitive decline and masticatory dysfunction. Alternatively, we reasoned that the decline in both cognitive and masticatory functions may be separately associated with deficits in other brain regions. We hypothesized that functional and structural changes in the cerebellum may play a key role to both cognitive decline and masticatory dysfunction in elderly individuals (Fig. 1c). Cerebellar atrophy is associated with poor cognitive performance [88] and predictive of the differentiation of Alzheimer’s and Parkinson’s dementia [89]. Cerebellar activation is frequently observed during chewing tasks [63, 66, 69, 70]. The intrinsic functional connectivity of the cerebellum with motor regions was found to be associated with masticatory performance [72]. A recent study that compared the brain signatures between the physically frail and non-frail elderly individuals revealed that the gray matter volume in the cerebellum was associated with the motor-related index of physical frailty [90]. Atrophy of the cerebellar Crus I and Crus II (Fig. 1c) was observed to be specific to Alzheimer’s disease and frontotemporal dementia [91]. Critically, the cerebellum plays a key role in age-related declines in cognitive and motor functions [92]. These findings suggest that a pre-existing deficit in critical brain regions, such as the cerebellum, may predispose individuals to functional declines in both mastication and cognition.

### Limitations: Beyond the brain-stomatognathic axis

The abovementioned hypotheses focused on the brain mechanisms that underlie the brain-stomatognathic axis. We limited our search to recent studies that focused on the relevant brain and stomatognathic mechanisms. However, it should be noted that other factors may play key roles in the association between masticatory dysfunction and cognitive decline. (A) First, both tooth loss or poor masticatory performance are related to oral hygiene skills and behavior, which may be compromised in the elderly individuals with cognitive impairment [11]. (B) Second, nutritional biomarkers (e.g., cholesterol) were reported to be independent risk markers of cognitive decline [93], and in elderly individuals, masticatory ability may explain part of the variance in nutrient intake [94]. (C) Third, among the major causes of tooth loss are periodontal diseases, which are associated with inflammation related to periodontal pathogens. The inflammatory damage of small blood vessels may play a key role in the pathogenesis of Alzheimer’s disease dementia [95]. Therefore, microbiological and immunological aspects, particularly periodontal conditions, should be considered [11, 13]. (D) Evidence from animal research has revealed that chewing may mediate the hippocampus-dependent cognitive deficit by suppressing HPA axis hyperactivity in the hippocampus [20, 80]. These considerations indicate that an integrative, multi-disciplinary investigation –including behavioral, nutritional, immunological and hormonal research – is necessary for a full understanding of the brain-stomatognathic axis.

### Considerations for future research

Based on the current evidence from the clinical, epidemiological, animal, and neuroimaging studies, we argue that the mechanisms underlying the brain-stomatognathic axis have not been elucidated, and the cause-and-effect relationship between cognitive decline and masticatory dysfunction requires more investigation. We suggest that the following aspects of this field must be highlighted in future research:

#### Refinement of behavioral assessments

In terms of ‘masticatory dysfunction’ most recent studies focused only on the anatomical deficit of the stomatognathic system, using the number of missing teeth as an index. Again, the anatomical deficit does not necessarily reflect the subjective chewing experience or objective masticatory performance. A more comprehensive assessment of the specific elements of stomatognathic function (e.g., masticatory performance, biting force, oral stereognosis, and masticatory muscles) and their interaction should be considered in future research. In terms of ‘cognitive decline’, our review revealed a substantial gap between the cognitive abilities assessed in human subjects and those assessed in animal research. A better reconciliation of the assessments between different species would increase the validity of the conclusions drawn from animal research.

#### Inclusion of baseline changes in mental and physical conditions

As revealed by animal research, the effect of masticatory dysfunction on cognitive decline may interact with the nutritional [46, 59] and mental condition of the organism [47, 52]. Notably, these factors are associated with the physical fitness of the elderly, and the interactional effect of these factors on cognitive decline should be considered.

#### Experimental designs that include prospective, longitudinal observations

One of the key requirements for understanding the mechanisms the brain-stomatognathic axis is the ability to differentiate between normal and pathological aging. The effect of long-term alterations, such as neuroplasticity effects in the brain, cannot be inferred from cross-sectional findings. A systematic collection of the results from masticatory and cognitive assessments would help clarify the effect of normal aging.

#### A precise estimation of the effect size

From a clinical perspective, the critical question may not always be to determine ‘If X and Y is associated’. Rather, to make a proper diagnosis and prognosis, clinicians need to know ‘To what extent does the change in X contribute the change in Y’, i.e., an estimation of the effect size of the association. As shown in a recent meta-analysis, suboptimal dentition (i.e., having <20 teeth) was associated with a 20% higher risk of cognitive decline [9, 52]. However, it is unclear if this is a pure effect from tooth loss or a combined effect due to other confounding factors. To estimate the actual effect that masticatory dysfunction has on cognitive decline is a clinically significant question.

## Conclusions

By revisiting recent evidence regarding the association between cognitive decline and masticatory dysfunction, we argue that the mechanisms underlying the brain-stomatognathic axis have not been fully elucidated. Moreover, it is concluded that the cause-and-effect relationship between cognitive decline and masticatory dysfunction requires more investigation. Three potential models and hypotheses of the brain-stomatognathic axis, focusing on the sensory feedback mechanisms, the compensation of motor control, and the cerebellum deficits, were summarized. Brain neuroimaging may contribute to identify the mechanisms underlying the brain-stomatognathic axis.

## Additional file

Additional file 1: Detailed procedures for systematic review and a list of the articles included in the review. (DOCX 46 kb)

## Abbreviations

APP: Amyloid precursor protein
BDNF: Brain-derived neurotrophic factor
CA: Cornu ammonis
CB: The cerebellum
CG: The cingulate gyrus
CS: Cross-sectional studies
dACC: The dorsal anterior cingulate cortex
DG: Dentate gyrus
FC: Functional connectivity
FG: The frontal gyrus
HIP: Hippocampus
HPA: Hypothalamic–pituitary–adrenal
INS: The insula
M1: The primary motor cortex
MRI: Magnetic resonance imaging
OB: Olfactory bulb
PFC: The prefrontal cortex
PMC: The premotor cortex
Pr5: The principal sensory trigeminal nucleus
PT: Prospective studies
RT: Retrospective studies
S1/S2: Primary/secondary somatosensory cortex
SMA: Supplementary motor area
SVZ: Subventricular zone
