# Supplementary material for: Revisiting the link between cognitive decline and masticatory dysfunction
Source: BMC Geriatr. 2018 Jan 5;18:5. doi: 10.1186/s12877-017-0693-z (PMC5756393; doi:10.1186/s12877-017-0693-z)
Supplement: Additional file 1: — Detailed procedures for systematic review and a list of the articles included in the review. (DOCX 46 kb) [file 12877_2017_693_MOESM1_ESM.docx]

**Revisiting the link between cognitive decline and masticatory dysfunction**

**Additional file 1:** Detailed procedures for systematic review and a list of the articles included in the review.

**Conclusion from the systematic review and meta-analysis on clinical and epidemiological research published in the past five years**

We adopted the following search criteria by investigating PubMed: (1) Key word combination: (chewing OR masticat* OR "tooth loss" OR "teeth loss" OR “number of teeth”) AND (cognit* OR memory OR dement*) AND ("systematic review" OR meta-analysis), (2) Language: English, (3) Publication date: within five years (2012.10.15 – 2017.10.15). This original search derived 12 articles (for a detailed article list, please see Table 1 of the Additional File). The articles were screened by the author (C-S Lin) for their eligibility. Seven articles were excluded for the following reasons: (a) being a secondary report (Kaye, 2017) for another original study (Cerutti-Kopplin et al., 2017), (b) focusing on the patients other than cognitive impairment or dementia (Schimmel et al., 2017; Osborne et al., 2017; Hirano & Onozuka, 2015), (c) focusing on the outcomes other than masticatory functions or tooth loss (Fleming et al., 2016; Farooqi et al., 2015; Tamura et al., 2013), and (d) the original full text was not acquired (Shen et al., 2016).

**Findings from the clinical / epidemiological research that objectively quantified masticatory performance using functional assessments published in the past five years**

We adopted the following search criteria by investigating PubMed: (1) Key word combination: ("mixing ability" OR "cutting ability" OR "crushing ability" OR "masticatory ability" OR "chewing ability" OR "masticatory performance" OR "masticatory efficiency" OR "chewing efficiency" OR "chewing performance") AND (cognit* OR memory OR dement*), (2) Language: English, (3) Publication date: within five years (2012.10.15 – 2017.10.15). This original search derived 23 articles (for a detailed article list, please see Table 1 of the Additional File). The articles were screened by the author (C-S Lin) for their eligibility. Eighteen articles were excluded for the following reasons: (a) focusing on the patients other than cognitive impairment or dementia (Inamochi et al., 2017; Schimmel et al., 2017; Komagamine et al., 2016; Lin et al., 2016; Listl et al., 2014; Weijenberg et al., 2013), (b) not an original research (Müller et al., 2017; Tarkowska et al., 2017; Douma et al., 2016; Muller, 2015; Savikko et al., 2013; Weijenberg et al., 2013). (c) focusing on the oral factors other than mastication (Lobbezoo et al., 2017), (d) only self-reported data of masticatory performance available (Park & Hong, 2017; Gellacic et al., 2016; Cerutti-Kopplin et al., 2015; Listl, 2014), (e) focusing only on disease-related issues (Lin et al., 2014)

**Findings from the animal research published in the past five years**

We adopted the following search criteria by investigating PubMed: (1) Key word combination: (chewing OR masticat* OR "tooth loss" OR "teeth loss") AND (hippocamp* OR parahippcamp* OR limbic), (2) Language: English, (3) Publication date: within five years (2012.10.15 – 2017.10.15). This original search derived 41 articles (for a detailed article list, please see Table 1 of the Additional File). The articles were screened by the author (C-S Lin) for their eligibility. Twenty-three articles were excluded for the following reasons: (a) non-animal research (Feng et al., 2017; Junming et al., 2014; Teixeira et al., 2014; Hansson et al., 2013), (b) not relevant to chewing movement (Mousavi et al., 2017; Kumar et al., 2017; Ohara et al., 2016; Bakhshishayan et al., 2013), (c) not an original research (Azuma et al., 2017; Kitz et al., 2017; Kubo et al., 2015; Chen et al., 2015; Klineberg et al., 2014), (d) not directly intervening the masticatory function (Kim et al., 2017; Hooshmandi et al., 2017; Kreiss & De Deurwaerdère, 2017; Kamyar et al., 2016; Kamada et al., 2013; Su et al., 2013), (e) focusing only on pain-related issues (Ding et al., 2016; Simonic-Kocijan et al., 2013), (f) focusing only on the cellular and neuronal changes on the offspring (Suzuki et al., 2016; Onishi et al., 2014)

**Findings from brain neuroimaging studies related to chewing published in the past five years**

We adopted the following search criteria by investigating PubMed: (1) Key word combination: (chewing OR masticat* OR "tooth loss" OR "teeth loss") AND MRI AND brain, (2) Language: English, (3) Publication date: within five years (2012.10.15 – 2017.10.15). This original search derived 54 articles (for a detailed article list, please see Table 1 of the Additional File). The articles were screened by the author (C-S Lin) for their eligibility. Forty-one articles were excluded for the following reasons: (a) not an human MRI study (Avivi-Arber et al., 2017; Yuan et al., 2014; Fukushima et al., 2014), (b) not relevant to the assessment of chewing movement (Custead et al., 2017; Moon et al., 2017; Kumar et al., 2017; Nguyen et al., 2016; Baharith & Zarrin., 2016; De Winter et al., 2016; Breshears et al., 2015; Meulman  et al., 2015; Bana et al., 2015; Scheuerle & Ursini, 2015; Kim et al., 2015; De Winter et al., 2015; Jacobs et al., 2014; Wadman et al., 2014; Meier et al., 2014; Jagota et al., 2014; Junming et al., 2014; Vanderver et al., 2014; Sperber et al., 2014; Taguchi et al., 2013; Garmi et al., 2013; Nowinski et al., 2013; Zhu et al., 2013), (c) focusing only on occlusion, clenching or bruxism (Feng et al., 2017; Yılmaz, 2015; Iida et al., 2014; Lotze et al., 2012), (d) focusing only on disease-related issues (Suenaga et al., 2016; Park et al., 2014; Kim et al., 2013; Vancaester et al., 2013; Mariën et al., 2013), (e) focusing only on the group difference (Yuan et al., 2017; Weng et al., 2017; Liu et al., 2016; Huang et al., 2017), (f) performing gum chewing with hand movement (Jang et al., 2015), (g) not an original research (Schreckenbach et al., 2014)

Table 1. Full list of the articles screened for eligibility

| **Conclusion from the systematic review and meta-analysis on clinical and epidemiological research published in the past five years** | | | | |
| --- | --- | --- | --- | --- |
| Title | First author | Year | | Source |
| Intoxication and substance use disorder to Areca catechu nut containing betel quid: A review of epidemiological evidence, pharmacological basis and social factors influencing quitting strategies. | Osborne PG | 2017 | | Drug Alcohol Depend |
| Limited Evidence Suggests Tooth Loss is Associated With Increased Risk of Cognitive Impairment. | Kaye EK. | 2017 | | J Evid Based Dent Pract |
| Periodontal disease, tooth loss and dementia: Is there a link? A systematic review. | Tonsekar PP | 2017 | | Gerodontology |
| Oro-facial impairment in stroke patients. | Schimmel M | 2017 | | J Oral Rehabil |
| Association between mastication and cognitive status: A systematic review. | Tada A | 2017 | | Arch Gerontol Geriatr |
| Oral health and orofacial pain in older people with dementia: a systematic review with focus on dental hard tissues. | Delwel S | 2017 | | Clin Oral Investig |
| Association Between Oral Health and Cognitive Status: A Systematic Review. | Wu B | 2016 | | J Am Geriatr Soc |
| Non-pharmacological interventions for alleviating pain during orthodontic treatment. | Fleming PS | 2016 | | Cochrane Database Syst Rev |
| Appropriate Recall Interval for Periodontal Maintenance: A Systematic Review. | Farooqi OA | 2015 | | J Evid Based Dent Pract |
| Association between tooth loss and dementia among older people: a meta-analysis. | Shen T | 2016 | | Int J Geriatr Psychiatry |
| Chewing and attention: a positive effect on sustained attention. | Hirano Y | 2015 | | Biomed Res Int |
| Factors associated with weight loss, low BMI, and malnutrition among nursing home patients: a systematic review of the literature. | Tamura BK | 2013 | | J Am Med Dir Assoc |
| **Findings from the clinical / epidemiological research that objectively quantified masticatory performance using functional assessments published in the past five years** | | | | |
| Title | First author | Year | | Source |
| Oral health for an ageing population: the importance of a natural dentition in older adults | Müller F | 2017 | | Int Dent J |
| Relationship between chewing ability and cognitive impairment in the rural elderly. | Kim EK | 2017 | | Arch Gerontol Geriatr |
| Oro-facial impairment in stroke patients. | Schimmel M | 2017 | | J Oral Rehabil |
| Combined effect of new complete dentures and simple dietary advice on nutritional status in edentulous patients: study protocol for a randomized controlled trial. | Komagamine Y | 2016 | | Trials |
| Correlation of cognitive and masticatory function in Alzheimer's disease. | Campos CH | 2017 | | Clin Oral Investig |
| Assessment of masticatory performance by means of a color-changeable chewing gum. | Tarkowska A | 2017 | | J Prosthodont Res |
| Gray Matter Volume and Resting-State Functional Connectivity of the Motor Cortex-Cerebellum Network Reflect the Individual Variation in Masticatory Performance in Healthy Elderly People. | Lin CS | 2015 | | Front Aging Neurosci |
| Oral mixing ability and cognition in elderly persons with dementia: a cross-sectional study. | Weijenberg RA | 2015 | | J Oral Rehabil |
| Interventions for edentate elders--what is the evidence? | Müller F | 2014 | | Gerodontology |
| Tooth loss, chewing efficiency and cognitive impairment in geriatric patients. | Elsig F | 2015 | | Gerodontology |
| Two-colour chewing gum mixing ability: digitalisation and spatial heterogeneity analysis. | Weijenberg RA | 2013 | | J Oral Rehabil |
| Adaptive change in chewing-related brain activity while wearing a palatal plate: an functional magnetic resonance imaging study. | Inamochi Y | 2017 | | J Oral Rehabil |
| Orofacial Pain and Mastication in Dementia. | Lobbezoo F | 2017 | | Curr Alzheimer Res |
| Predictors of chewing ability among community-residing older adults in Korea. | Park K | 2017 | | Geriatr Gerontol Int |
| The effects of video observation of chewing during lunch on masticatory ability, food intake, cognition, activities of daily living, depression, and quality of life in older adults with dementia: a study protocol of an adjusted randomized controlled trial. | Douma JG | 2016 | | BMC Geriatr |
| Cognitive status of edentate elders wearing complete denture: Does quality of denture matter? | Cerutti-Kopplin D | 2015 | | J Dent |
| Factors associated with deterioration of self-rated chewing ability among adults aged 60 years and older over a 6-year period. | Gellacic AS | 2016 | | Geriatr Gerontol Int |
| Are early onset aging conditions correlated to daily activity functions in youth and adults with Down syndrome? | Lin JD | 2014 | | Res Dev Disabil |
| Oral health conditions and cognitive functioning in middle and later adulthood. | Listl S | 2014 | | BMC Oral Health |
| Early life conditions, adverse life events, and chewing ability at middle and later adulthood. | Listl S | 2014 | | Am J Public Health |
| Chewing ability and dementia. | Savikko N | 2013 | | J Am Geriatr Soc |
| Increased masticatory activity and quality of life in elderly persons with dementia--a longitudinal matched cluster randomized single-blind multicenter intervention study. | Weijenberg RA | 2013 | | BMC Neurol |
| Evaluation of chewing ability and its relationship with activities of daily living, depression, cognitive status and food intake in the community-dwelling elderly. | Kimura Y | 2013 | | Geriatr Gerontol Int |
|  |  |  | |  |
| **Findings from the animal research published in the past five years** | | | | |
| Title | First author | | Year | Source |
| Brain activation patterns during unilateral premolar occlusion. | Feng CZ | | 2017 | Cranio |
| Functional connectivity of the hippocampus to the thalamocortical circuitry in an animal model of absence seizures. | Mousavi SR | | 2017 | Epilepsy Res |
| Association between Mastication, the Hippocampus, and the HPA Axis: A Comprehensive Review. | Azuma K | | 2017 | Int J Mol Sci |
| Decreased hippocampal brain-derived neurotrophic factor and impaired cognitive function by hypoglossal nerve transection in rats. | Kim D | | 2017 | J Cell Mol Med |
| Reduced Mastication Impairs Memory Function. | Fukushima- Nakayama Y | | 2017 | J Dent Res |
| Antagonism of orexin type-1 receptors (OX1Rs) attenuates naloxone-precipitated morphine withdrawal syndrome in rat dorsal hippocampus. | Hooshmandi M | | 2017 | Pharmacol Biochem Behav |
| Purposeless oral activity induced by meta-chlorophenylpiperazine (m-CPP): Undefined tic-like behaviors? | Kreiss DS | | 2017 | J Neurosci Methods |
| Feline Temporal Lobe Epilepsy: Review of the Experimental Literature. | Kitz S | | 2017 | J Vet Intern Med |
| The Brain Basis for Misophonia. | Kumar S | | 2017 | Curr Biol |
| Widespread Volumetric Brain Changes following Tooth Loss in Female Mice. | Avivi-Arber L | | 2016 | Front Neuroanat |
| Molar loss and powder diet leads to memory deficit and modifies the mRNA expression of brain-derived neurotrophic factor in the hippocampus of adult mice. | Takeda Y | | 2016 | BMC Neurosci |
| Crocin prevents haloperidol-induced orofacial dyskinesia: possible an antioxidant mechanism. | Kamyar M | | 2016 | Iran J Basic Med Sci |
| Tooth loss early in life suppresses neurogenesis and synaptophysin expression in the hippocampus and impairs learning in mice. | Kubo KY | | 2017 | Arch Oral Biol |
| Maternal chewing during prenatal stress ameliorates stress-induced hypomyelination, synaptic alterations, and learning impairment in mouse offspring. | Suzuki A | | 2016 | Brain Res |
| Direct projection from the lateral habenula to the trigeminal mesencephalic nucleus in rats. | Ohara H | | 2016 | Brain Res |
| Hippocampus-dependent spatial memory impairment due to molar tooth loss is ameliorated by an enriched environment. | Kondo H | | 2016 | Arch Oral Biol |
| Zinc deficiency with reduced mastication impairs spatial memory in young adult mice. | Kida K | | 2015 | Physiol Behav |
| Mastication as a Stress-Coping Behavior. | Kubo KY | | 2015 | Biomed Res Int |
| Chewing Maintains Hippocampus-Dependent Cognitive Function. | Chen H | | 2015 | Int J Med Sci |
| Chewing prevents stress-induced hippocampal LTD formation and anxiety-related behaviors: a possible role of the dopaminergic system. | Ono Y | | 2015 | Biomed Res Int |
| Inflammatory pain memory facilitates occlusal interference-induced masticatory muscle hyperalgesia in rats. | Ding TT | | 2016 | Eur J Pain |
| Behavioral impairments and changes of nitric oxide and inducible nitric oxide synthase in the brains of molarless KM mice. | Pang Q | | 2015 | Behav Brain Res |
| Tooth loss inhibits neurogenesis in the dentate gyrus of adult mice. | Su S | | 2014 | Neural Regen Res |
| Tooth loss might not alter molecular pathogenesis in an aged transgenic Alzheimer's disease model mouse. | Oue H | | 2016 | Gerodontology |
| Contemporary relevance of occlusion and mastication. | Klineberg I | | 2014 | Int J Prosthodont |
| Hard-diet feeding recovers neurogenesis in the subventricular zone and olfactory functions of mice impaired by soft-diet feeding. | Utsugi C | | 2014 | PLoS One |
| Liquid diet induces memory impairment accompanied by a decreased number of hippocampal neurons in mice. | Okihara H | | 2014 | J Neurosci Res |
| Anterior corpus callosotomy combined with anterior temporal resection with amygdalohippocampectomy: outcome in a patient with congenital bilateral perisylvian syndrome. | Junming Z | | 2014 | Turk Neurosurg |
| Masticatory deficiency as a risk factor for cognitive dysfunction. | Teixeira FB | | 2014 | Int J Med Sci |
| Soft-diet feeding after weaning affects behavior in mice: Potential increase in vulnerability to mental disorders. | Nose-Ishibashi K | | 2014 | Neuroscience |
| Loss of molars early in life develops behavioral lateralization and impairs hippocampus-dependent recognition memory. | Kawahata M | | 2014 | BMC Neurosci |
| TRPV1 channel-mediated bilateral allodynia induced by unilateral masseter muscle inflammation in rats. | Simonic- Kocijan S | | 2013 | Mol Pain |
| Learning deficits and suppression of the cell proliferation in the hippocampal dentate gyrus of offspring are attenuated by maternal chewing during prenatal stress. | Onishi M | | 2014 | Neurosci Lett |
| Protein kinase A regulates the long-term potentiation of intrinsic excitability in neonatal trigeminal motoneurons. | Bakhshishayan S | | 2013 | Brain Res |
| Liquid diets reduce cell proliferation but not neurogenesis in the adult rat hippocampus. | Patten AR | | 2013 | Neuroscience |
| Influence of a long-term powdered diet on the social interaction test and dopaminergic systems in mice. | Niijima-Yaoita F | | 2013 | Neurochem Int |
| Relationship between natural teeth and memory in a healthy elderly population. | Hansson P | | 2013 | Eur J Oral Sci |
| Tooth loss induces memory impairment and neuronal cell loss in APP transgenic mice. | Oue H | | 2013 | Behav Brain Res |
| Spontaneous seizures in a rat model of multiple prenatal freeze lesioning. | Kamada T | | 2013 | Epilepsy Res |
| Forced mastication increases survival of adult neural stem cells in the hippocampal dentate gyrus. | Akazawa Y | | 2013 | Int J Mol Med |
| Aquaporin-4 knockout abolishes apomorphine-induced tardive dyskinesia following chronic treatment with neuroleptics. | Su CJ | | 2012 | CNS Neurosci Ther |
| **Findings from brain neuroimaging studies related to chewing published in the past five years** | | | | |
| Title | First author | | Year | Source |
| Brain encoding of saltatory velocity through a pulsed pneumotactile array in the lower face. | Custead R | | 2017 | Brain Res |
| Brain activation patterns during unilateral premolar occlusion. | Feng CZ | | 2017 | Cranio |
| Altered Gray-Matter Volumes Associated With Betel Quid Dependence. | Yuan F | | 2017 | Front Psychiatry |
| Adaptive change in chewing-related brain activity while wearing a palatal plate: an functional magnetic resonance imaging study. | Inamochi Y | | 2017 | J Oral Rehabil |
| The brain activation pattern of the medial temporal lobe during chewing gum: a functional MRI study. | Choi YH | | 2017 | Neural Regen Res |
| Age-Related Difference in Functional Brain Connectivity of Mastication. | Lin CS | | 2017 | Front Aging Neurosci |
| The usefulness of diagnostic imaging for the assessment of pain symptoms in temporomandibular disorders. | Suenaga S | | 2016 | Jpn Dent Sci Rev |
| Periventricular White Matter Lesions as a Prognostic Factor of Swallowing Function in Older Patients with Mild Stroke. | Moon HI | | 2017 | Dysphagia |
| Evaluation of structural connectivity changes in betel-quid chewers using generalized q-sampling MRI. | Weng JC | | 2017 | Psychopharmacology (Berl) |
| The Brain Basis for Misophonia. | Kumar S | | 2017 | Curr Biol |
| Widespread Volumetric Brain Changes following Tooth Loss in Female Mice. | Avivi-Arber L | | 2016 | Front Neuroanat |
| Gliosarcoma with Primary Skull Base Invasion. | Nguyen QD | | 2016 | Case Rep Radiol |
| Altered Long- and Short-Range Functional Connectivity in Patients with Betel Quid Dependence: A Resting-State Functional MRI Study. | Liu T | | 2016 | Cell Physiol Biochem |
| Khat - a new precipitating factor for reversible cerebral vasoconstriction syndrome: a case report. | Baharith H | | 2016 | J Med Case Rep |
| Amygdala atrophy affects emotion-related activity in face-responsive regions in frontotemporal degeneration. | De Winter F | | 2016 | Cortex |
| Betel quid chewing alters functional connectivity in frontal and default networks: A resting-state fMRI study. | Huang X | | 2017 | J Magn Reson Imaging |
| Symmetry of fMRI activation in the primary sensorimotor cortex during unilateral chewing. | Lotze M | | 2017 | Clin Oral Investig |
| Gray Matter Volume and Resting-State Functional Connectivity of the Motor Cortex-Cerebellum Network Reflect the Individual Variation in Masticatory Performance in Healthy Elderly People. | Lin CS | | 2015 | Front Aging Neurosci |
| Mastication induces long-term increases in blood perfusion of the trigeminal principal nucleus. | Viggiano A | | 2015 | Neuroscience |
| The cortical effect of chewing gum during hand movements: A functional MRI study. | Jang SH | | 2015 | Somatosens Mot Res |
| A probabilistic map of the human ventral sensorimotor cortex using electrical stimulation. | Breshears JD | | 2015 | J Neurosurg |
| Ischemic cardiomyopathy and cerebral infarction in a young patient associated with khat chewing. | Meulman TJ | | 2015 | Case Rep Radiol |
| To see bruxism: a functional MRI study. | Yılmaz S | | 2015 | Dentomaxillofac Radiol |
| A Case of Masticatory Dystonia Following Cerebellar Haemorrhage. | Bana C | | 2015 | Cerebellum |
| Incontinentia Pigmenti. | Scheuerle AE | | 1993 | GeneReviews® |
| Brain networks engaged in audiovisual integration during speech perception revealed by persistent homology-based network filtration. | Kim H | | 2015 | Brain Connect |
| Lateralization for dynamic facial expressions in human superior temporal sulcus. | De Winter FL | | 2015 | Neuroimage |
| Collateral circulation prevents masticatory muscle impairment in rat middle cerebral artery occlusion model. | Yuan F | | 2014 | J Synchrotron Radiat |
| Increased coupling of intrinsic networks in remitted depressed youth predicts rumination and cognitive control. | Jacobs RH | | 2014 | PLoS One |
| Analysis of brain activity involved in chewing-side preference during chewing: an fMRI study. | Jiang H | | 2015 | J Oral Rehabil |
| Bulbar muscle MRI changes in patients with SMA with reduced mouth opening and dysphagia. | Wadman RI | | 2014 | Neurology |
| Equal pain-Unequal fear response: enhanced susceptibility of tooth pain to fear conditioning. | Meier ML | | 2014 | Front Hum Neurosci |
| An electrocorticographic electrode array for simultaneous recording from medial, lateral, and intrasulcal surface of the cortex in macaque monkeys. | Fukushima M | | 2014 | J Neurosci Methods |
| Transplacental transfer of NMDA receptor antibodies in an infant with cortical dysplasia. | Jagota P | | 2014 | Neurology |
| Influence of posterior dental arch length on brain activity during chewing in patients with mandibular distal extension removable partial dentures. | Shoi K | | 2014 | J Oral Rehabil |
| Anterior corpus callosotomy combined with anterior temporal resection with amygdalohippocampectomy: outcome in a patient with congenital bilateral perisylvian syndrome. | Junming | | 2014 | Turk Neurosurg |
| Leukodystrophy Overview. | Vanderver A | | 1993 | GeneReviews® |
| The genesis of craniofacial biology as a health science discipline. | Sperber GH | | 2014 | Aust Dent J |
| Analysis of brain and muscle activity during low-level tooth clenching--a feasibility study with a novel biting device. | Iida T | | 2014 | J Oral Rehabil |
| Mastication-induced vertigo and nystagmus. | Park SH | | 2014 | J Neurol |
| Novel TPM3 mutation in a family with cap myopathy and review of the literature. | Schreckenbach T | | 2014 | Neuromuscul Disord |
| Association between oral health and the risk of lacunar infarction in Japanese adults. | Taguchi A | | 2013 | Gerontology |
| Neuroplasticity in the adaptation to prosthodontic treatment. | Luraschi J | | 2013 | J Orofac Pain |
| Pure motor trigeminal neuropathy in a woman with tegmental pontine infarction. | Kim DH | | 2013 | J Clin Neurosci |
| Masticatory myorhythmia following pontine hemorrhage. | Vancaester E | | 2013 | Acta Neurol Belg |
| Lengthening temporalis myoplasty and brain plasticity: a functional magnetic resonance imaging study. | Garmi R | | 2013 | Ann Chir Plast Esthet |
| Gum chewing inhibits the sensory processing and the propagation of stress-related information in a brain network. | Yu H | | 2013 | PLoS One |
| Three-dimensional interactive and stereotactic atlas of head muscles and glands correlated with cranial nerves and surface and sectional neuroanatomy. | Nowinski WL | | 2013 | J Neurosci Methods |
| Effects of chewing on cognitive processing speed. | Hirano Y | | 2013 | Brain Cogn |
| Functional connectivity of human chewing: an fcMRI study. | Quintero A | | 2013 | J Dent Res |
| Dissimilar processing of emotional facial expressions in human and monkey temporal cortex. | Zhu Q | | 2013 | Neuroimage |
| Brain activity and human unilateral chewing: an FMRI study. | Quintero A | | 2013 | J Dent Res |
| Mastication dyspraxia: a neurodevelopmental disorder reflecting disruption of the cerebellocerebral network involved in planned actions. | Mariën P | | 2013 | Cerebellum |
| The cerebral representation of temporomandibular joint occlusion and its alternation by occlusal splints. | Lotze M | | 2012 | Hum Brain Mapp |
